# Validated Densitometric TLC-Method for the Simultaneous Analysis of (*R*)- and (*S*)-Citalopram and its Related Substances Using Macrocyclic Antibiotic as a Chiral Selector: Application to the Determination of Enantiomeric Purity of Escitalopram

**Published:** 2012-03

**Authors:** Suzan Mahmoud Soliman

**Affiliations:** *National Organization for Drug Control and Research (NODCAR), Egypt*

**Keywords:** enantiomers, (*R*)- and (*S*)-citalopram, norvancomvcin, related substance, TLC-determination, pharmaceutical preparations

## Abstract

A novel economic procedure for the simultaneous stereospecific separation and analysis of (*R*)- and (*S*)-citalopram and its related substances or impurities has been developed and validated. Chromatography was performed on silica gel 60 F254 plates with acetonitrile: methanol: water (15:2.5:2.5: v/v/v) as a mobile phase containing 1.5 mM norvancomycin or 2.5 mM vancomycin as a selector at ambient temperature. (*R*)- and (*S*)-citalopram enantiomers in presence of its related substances; citalopram citadiol and citalopram N-oxide were well separated with significant Rf values of 0.33 ± 0.02, 0.85 ± 0.02, 0.45 ± 0.02 and 0.22 ± 0.02, respectively. The spots were detected with either iodine vapor, or by use of a UV lamp followed by densitometric measurement at 239 nm. All variables affecting the resolution, such as concentration of chiral selectors, mobile phase system at different temperatures and pH-values were investigated and the conditions were optimized. Calibration plots for analysis of (*R*)- and (*S*)-enantiomers were linear in the range of 0.2-16.8 μg/10 μl (R≥0.9994, n=6) with acceptable precision (%RSD<2.0) and accuracy (99.70 ± 0.85% and 99.51 ± 0.61% for (*S*)-citalopram and escitalopram, respectively). The limit of detection and quantification were 0.08 μg/10 μl and 0.25 μg/10 μl, respectively, for (*R*)- and (*S*)-citalopram. The proposed method is simple, selective, and robust and can be applied for quantitative determination of enantiomeric purity of (*R*)- and (*S*)-citalopram (escitalopram) as well as the related impurities in drug substances and pharmaceutical preparations. The method can be useful to investigate adulteration of pure isomer with the cheep racemic form.

## INTRODUCTION

Chiral discrimination has been an issue in the development and use of pharmaceutical drugs because enantiomers may have various pharmacokinetic properties and produce different responses. The administration of highly pure chiral drugs is a major goal of pharmaceutical industry to protect the client against strains caused by high drug concentration or toxic side effects.

The antidepressive drug citalopram (CIT) is selective serotonin reuptake inhibitors (SSRIs). Potentiation of serotonergic neurotransmission gives a broad spectrum of therapeutic activity in depression, anxiety, obsessional and impulse control disorders (1, 2).

Citalopram, (*R, S*)-CIT, is a chiral compound has one asymmetric carbon atom in the isobenzofuran ring. The pharmacological effect of (*R, S*)-CIT is related mainly to (*S*)-CIT, escitalopram (ESC). CIT is sold as a racemic mixture, consisting of 50 % *R*-(-)- CIT and of 50 % *S*-(-)- CIT. (*S*)-CIT enantiomer (ESC) has a pharmacological efficiency different than (*R*)-CIT. It inhibited serotonin functions approximately 100 fold more potent than (*R*)-CIT and at least 2 times more potently than racemic CIT. The lower efficacy of citalopram is due to inhibition of the pharmacological effect by the (*R*)-enantiomer (3-5). CIT is official in the United State Pharmacopoeia 34 and the British Pharmacopoeia 2011, both pharmacopoeias determined purity of the drug as racemic mixture by non-chiral HPLC-procedures without separation of the two enantiomers.

Owing to the existence of pharmacological and toxicological differences between stereoisomers, enantiomeric separation is now an integral part of drug research. Therefore, many chromatographic methods have been established for analysis of drug enantiomers. Among them so far developed, high performance liquid chromatography HPLC based on chiral stationary phase and capillary electrophoresis CE are widely employed for the assay of drug isomers in pharmaceutical preparations. Different chromatographic-methods have been reported for assay of CIT using different HPLC procedures (6-8) and CE (9, 10).

A literature survey revealed that limited TLC methods have been reported for analysis of (*R, S*)-CIT in pharmaceutical preparations. The reported method (11) describes HPTLC-procedure for the determination of citalopram in tablets. Stability-indicating HPTLC method has been developed for assay of (*S*)-CIT in presence of unknown degradation products formed through forced degradation studies (12), but it was out of scope because it did not separate and determine the impurities and has been established as non-chiral TLC-procedure (11, 12). It is worth noting that only one TLC- method has been reported for separation of CIT (13). In this method only one enantiomer (*S*-CIT) was determined (13). Moreover, none of the existing studies reported impurity details (11-13).

This study represents the first enantioselective TLC-method for separation of (*R, S*)-citalopram and its related substances using vancomycin or norvancomycin as chiral selector. Chiral recognition has been achieved through formation of host - a guest inclusion complex via attractive interactions. The analysis of the two enantiomers as well as the related substances was established. Compared with other chromatographic techniques TLC has advantages related to its simplicity of performance, low expense and wide use for running several samples simultaneously. This is very convenient not only for increased sample throughput but also because the positions of spots can be easily compared with those from reference samples running in parallel (14, 15). Norvancomycin or vancomycin has been used as a chiral selector (CS) in LC and CE (16, 17), as well as TLC method (18, 19).

**Figure 1:**
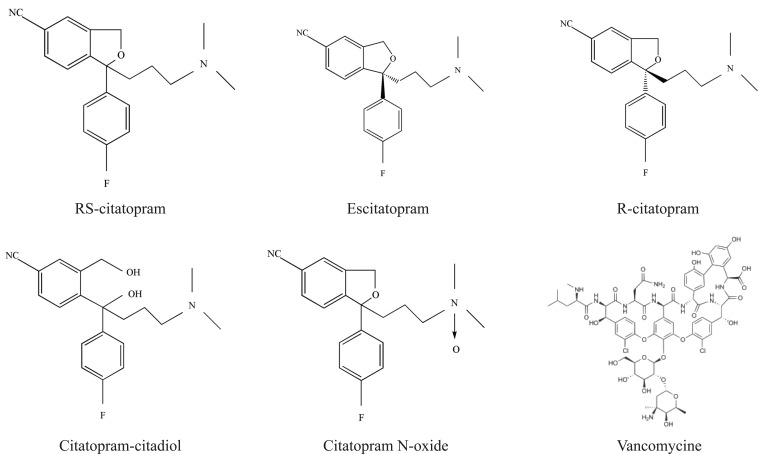
The structure of *R, S*-citalopram, escitalopram, related substances, and vancomycine as a chiral selector.

Norvancomycin (NORV), as an analogue antibiotic of VANC, lacks a methyl group in its chemical structure and consists of three fused macrocyclic rings, two side chains, a carbohydrate dimer and leucine (N-methyl-leucine in vancomycin) (Fig. 1), resulting in a notably higher chiral selectivity in chiral compounds containing a free carboxylic acid functional group (16).

Macrocyclic antibiotics have several stereogenic centers and functional groups allowing multiple interactions with chiral analytes. The glycopeptides consist of a carbohydrate moiety and semirigid basket-shaped aglycan. The hydrophobic properties of the CS enable the formation of host-guest inclusion complexes.

Armstrong and Zhou introduced VANC as a chiral selector for enantiomeric separation by TLC (18). Hydrogen bonds with the pendant arms as well as dipole stacking, ionic-, p–p interactions and steric repulsions are assumed to be the main interactions responsible for chiral recognition. In addition to ionic interactions, hydrogen bondings and p–p interaction were proposed as forces allowing multiple enantioselective interactions with the analytes (18).

The related substances are strictly similar in structure to the active ingredient. Citalopram citadiol (CIT-C) is (4-(4-dimethylamino)-1-(4-flurophenyl)-1-hydroxybutyl-3-(hydroxyl metyl)-benzonitrile (20). It is reported that it is the synthetic intermediate of ESC (21). Citalopram N-oxide (CIT-N), 1-[3-(dimethylamino) propyl]-1-(4-flurophenyl)-1, 3-dihydro-5-isobenzofuran carbonitrile N-oxide, is the active metabolite of the drug (22).

So, an enantioselective TLC assay appears a critical step in the development of high-quality manufacturing processes and quality control methods. The structure of (R, S)-CIT, escitalopram and their related substances as well as vancomycin are represented in Fig. 1.

Hence a simple reproducible direct enantiomeric TLC-method was developed and validated for quantitative determination of *R, S*-citalopram, escitalopram and related substances in raw materials and pharmaceutical dosage forms. Nor vancomycin or vancomycin has been used as a chiral selector (CS). Conditions affording best resolution were optimized and the method was validated as per USP guidelines (20).

## EXPERIMENTAL

### Instrumentation

Densitometer-Dual wavelength Flying spot-scanning, Shimadzu CS-9301 (Japan). UV lamp-short wavelength 254 nm (Japan). High performance thin-layer chromatographic plates-Silica Gel 60F254, 20 × 20 cm, 0.2 mm thickness, fluorescent at 254 nm (E. Merck, Germany). Jar-Glass, 20 × 20 × 10 cm.

UV-Vis Spectrophotometer- Shimadzu (Tokyo, Japan).

### Materials and reference samples

Citalopram hydrobromide working standard was kindly supplied by Adwia CO, Egypt; its purity was found to be 99.00 ± 1.13%, according to the reported method (13). Depram tablets (batch No. 1990709) labeled to contain 40 mg CIT per tablet and produced by Apex pharma, Egypt. Citalo tablets (batch No. 740127) nominally containing 20 mg CIT per tablet were kindly supplied from Delta pharma, Egypt.

Ecitalopram oxalate was used as reference standard, was kindly supplied by Genesis pharma, Egypt, Matrix laloratories Limitted; its purity was found to be 99.15 ± 1.08%, according to the reported method (13). Cipra-Pro tablets (batch No. 83292) contain 10 mg ECS per tablet were produced by Marcyrl Pharmaceutical Industries for Copad pharma, Egypt and kindly supplied by Genesis Pharma, Egypt, Matrix Laboratories Limited. Estikan tablets (batch No. 96585) contain 20 mg ECS per tablet and produced by Alhekma pharma, Egypt. Methanol, acetonitrile were from (Lab-Scan, Cairo, Egypt), ortho phosphoric acid and sodium hydroxide were from (Analytical grade, Merk, Darmstadt, Germany),

The related impurities; (*R*)-citalopram oxalate (*R*)-CIT, Citalopram citadiol (CIT- C), Citalopram N-oxide (CIT-N) were kindly supplied by Genesis pharma Egypt, Matrix Laboratories Limited. Chiral selectors; norvancomycin and vancomycin were from (Fluka, Egypt).

### Chromatographic Conditions

Chromatographic separation was achieved on TLC aluminum plates pre-coated with silica gel 60 F-254, (10 cm × 10 cm) with 0.2 mm thickness. 10 μl of each working solution was spotted from the bottom of the precoated TLC aluminum plates using a 25 μl Hamilton micro- syring. Cleaned and dried glass chromatographic chamber (6 × 12 × 12 cm) was pre-equilibrated with the developing mobile phase for 20 min. The plates were developed by ascending chromatography with mobile phase consisting of acetonitrile-methanol-water (15:2.5:2.5: v/v/v) containing 1.5 mM norvancomycin or 2.5 mM vancomycin as a chiral selector, at pH7-8. Linear ascending development was carried out to a distance of 8 cm at ambient temperature of 25 ± 2°C. The plates were air dried. The spots were detected with either iodine vapor, or by use of a UV lamp at 254 nm. TLC scanner was used for the analysis under zigzag scanning mode, the reflection-absorption mode at 239 nm and swing width 10 mm.

### Preparation of standard solutions

A standard solution of (*R, S*)-CIT (4 mg/ml) was prepared in methanol. Serial dilutions, in methanol, in a series of 5 ml volumetric flasks containing (0.04-3.36 mg/ml of CIT in methanol was used as working solutions for preparation of calibration curve.

A standard solution of ESC (2 mg/ml) was prepared in methanol. Serial dilutions, in methanol, in a series of 5-ml volumetric flasks containing (0.02-1.68 mg/ml of ESC in methanol was used as working solutions for preparation of calibration curve.

Standard solutions of each impurity (1 mg/ml) were prepared in methanol. Serial dilutions in two sets of 5 ml volumetric flasks each one containing 0.1-4.4 μg/10 μl of CIT-C or CIT-N in methanol were used as working solutions for preparation of corresponding calibration curves.

### General procedures and calibration graphs

An aliquot of 10 μl of working solution (0.04-3.36 mg/ml) of (R, S)-CIT, or (0.02-1.68 mg/ml) of (S)-CIT (escitalopram) and (0.01-0.44 mg/ml) each of the related substances was applied on the TLC plates. The procedure was carried out as mentioned under the chromatographic conditions. The calibration curve for each solution was obtained by plotting peak areas versus the corresponding concentrations of each studied compound.

### Assay of laboratory-prepared mixtures

The thin layer chromatogram was recorded for the five synthetic prepared mixtures. The densitometry measurement at 239 nm was used for analysis of the examined compounds by the proposed TLC method. The concentration ratios of the related substance relative to ESC in the mixtures were (0.05-150%) *w/w*. The concentration of each compound in the mixture was calculated as described under general procedures and calibration graphs.

### Assay of Depram and Estikan tablets

Ten tablets of each pharmaceutical preparation were weighed and thoroughly ground. An accurately weighed amount of the finely powdered (Depram tablets) or (Estikan tablets) equivalent to 200 mg or 100 mg of (*R, S*)-CIT or ESC, respectively, was transferred into two separate 50 ml volumetric flask, extracted with methanol and made up to 50 ml with the same volume. The solutions were filtered and accurately measured aliquots of each filtrate were transferred to separate 5 ml volumetric flasks, diluted to 5 ml with methanol to give a concentration range of (0.04-3.36 mg/ml) and (0.02-1.68 mg/ml), respectively. 10 μl of each standard solution was spotted to the TLC plates. The plates were chromatographed as previously described under general procedures and calibration graphs, and the densitometric peak areas for each drug were measured, and the results were recorded.

### Detection and quantification of the related substances

Six different commercial brands, two containing *R, S*-CIT and ESC bulk drugs and four containing *R, S*-CIT and ESC tablets, were investigated.

For the bulk powder; a standard solution of 20 and 10 mg/ml of (*R, S*)-CIT or ESC, respectively, was prepared in methanol, and termed as sample solution (a) (10 mg/ml of ESC or *S*-CIT).

For the tablets, a quantity of the powdered (Depram tablets, Citalo-tablets) or (Cipra-pro tablets, Estikan tablets) equivalent to (2 g) of (*R, S*)-CIT or (1 g) of ESC, respectively, was transferred into four separate 100 ml volumetric flask, extracted with methanol and made up to 100 ml with methanol, the solutions were filtered and termed as sample solution (b) (10 mg/ml of ESC or *S*-CIT)

Standard solutions of each related substance (1 mg/ml) were prepared in methanol. Dilutions in three sets of 10-ml volumetric flasks containing 0.2 mg/ml each of (RCIT, CIT-C and CIT-N) were termed as standard solutions (c).

Ten μl of both the sample solution (a), (b) and the standard solutions (c) were applied on the TLC plates, and the chromatograms were run as previously described under general procedures and calibration graphs, and the densitometric peak areas were measured.

The peak area of each related substance was identified and quantified by spotting and comparing with the R_f_ value of the standard peak.

## RESULTS AND DISCUSSION

TLC has made great progress and attained wide acceptance as a major analytical tool for both quantitative and qualitative analysis. The experimental conditions for the proposed method, such as concentration of NORV and VANC, mobile phase composition, mobile phase pH and Temperature, were optimized to provide accurate and reproducible results.

The enantioselective analysis of ESC in drug substance and pharmaceutical preparations is not officially recorded in any pharmacopoeia, and the data does not provide any information about the related substances or impurities. The related substances of ESC are the same to those reported in the USP monograph for the racemic form. In fact, the production of ESC has been achieved via preparative chromatographic enantioresolution of racemic citadiol. Thus, the chiral purity of ESC depends on the purity of starting citadiol. So, before developing an enantioselective method to be applied to analysis of ESC, the effort must be focused on enantioseparation and characterization of (*R, S*) CIT and its related substances or impurities.

The present work includes 1) TLC-technique for identification and determination of CIT and ESC; 2) generation of the standard calibration curves; 3) determination of both drugs in presence of their related substances; 4) quantitative analysis of the individual CIT, ESC in their pharmaceutical preparations; 5) detection and quantitation of the related substances relative to 10 mg/ml drug substances and pharmaceutical preparations.

### Effect of mobile phase composition

Both compositions of the mobile phase and the nature of chiral stationary phase additive are strongly influenced enantiomeric resolution. Acetonitrile was the organic modifier that produced the most effective separations with the shortest development times. The proposed TLC method was optimized on using acetonitrile - methanol - water (15:2.5:2.5) v/v containing 1.5 mM NORV (Table 1). To improve the resolution of both enantiomers small amount of water would be used in the solvent system. The attractive interaction of a hydrogen atom in water molecules must be covalently bonded to another electronegative atom in the mobile phase system such as: nitrogen, oxygen or fluorine to create hydrogen bonding (5 to 30 kJ mol^-1^) (23).

**Table 1:** Effect of mobile phase system on enantiomeric resolution of (*R, S*)-citalopram and the related

Mobile phase (A:B:C^a^)	h R_f_ values				(*R, S*)-citalopram		h R_f_ (S)/R_f_ R)
	Standard sample						
	S-CIT	R-CIT	CIT-C	CIT-N	*S*-form	*R*-form	

13:5.0:2.0	0.81	0.40	0.42	0.18	0.81	0.40	2.02
13:2.0:5.0	0.77	0.48	0.53	0.31	0.77	0.48	1.61
15:2.5:2.5	0.85	0.33	0.45	0.22	0.85	0.33	2.58
17:0.0:3.0	0.88	0.51	0.56	0.28	0.88	0.51	1.72

a Acetonitrile-methanol-water, containing 1.5 mM norvancomycin.

### Effect of temperature and pH

**Figure 2:**
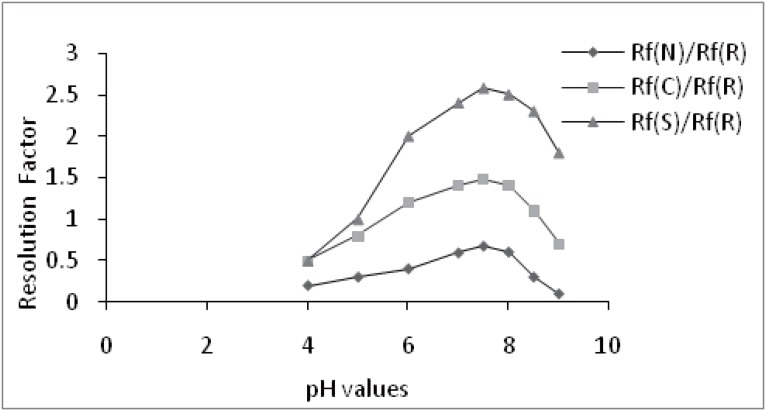
Effect of different pH values on relative retardation ratios Rret (resolution factor) on enantiomeric resolution of *R*-citalopram (*R*) and *S*-citalopram and the related substances; citalopram-N-oxide (N) and citalopram-citadiol (C).

In order to achieve enantioselectivity, the pH and temperature were studied and adjusted by addition of dilute phosphoric acid and/or dilute sodium hydroxide. At convenient pH and temperature hydrophobic parts of the analyte may be included into the hydrophobic basket of CS. Significant R_f_ values of the compounds was observed between pH7–8 (Fig. 2), at ambient temperature 25 ± 2°C, the data are given in Table 2.

**Table 2:** Effect of pH and temperature on the enantiomeric resolution of (*R, S*)-citalopram on TLC Plates using 1.5 mM vancomycin as achiral selector

Mobile phase system		h R_f_ values			hR_f_ (S)/hR_f_ (R)
Acetonitrile-methanol-water 15:2.5:2.5 (v/v)		Pure S-form	Racemic citalopram		
pH values	°C ± 2		R-form	S-form	

4-5	15	0.30	0.27	0.30	1.11
6-7	15	0.38	0.31	0.38	1.23
7-8	15	0.44	0.35	0.44	1.26
8-9	15	0.57	0.41	0.57	1.39
4-5	25	0.76	0.72	0.76	1.06
6-7	25	0.87	0.42	0.87	2.07
7-8	25	0.85	0.33	0.85	2.56
8-9	25	0.74	0.35	0.74	2.11
4-5	35	0.38	0.36	0.38	1.06
6-7	35	0.42	0.39	0.42	1.08
7-8	35	0.48	0.41	0.48	1.17
8-9	35	0.71	0.52	0.71	1.37

### Effect of norvancomycin as a chiral selector

**Figure 3:**
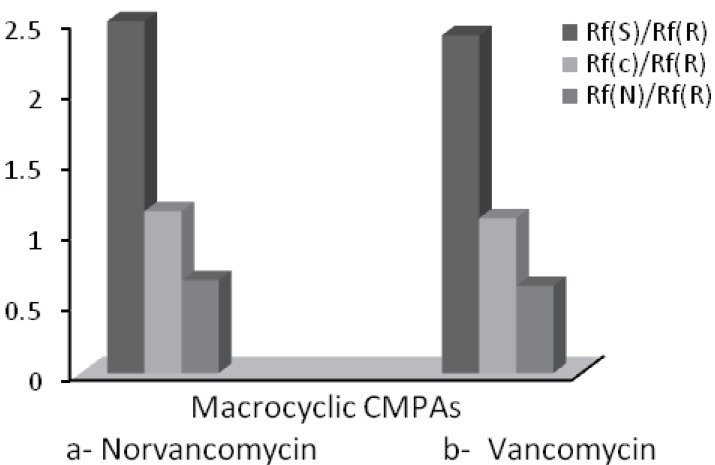
Effect of two different type of macrocyclic CMPAs. (a) Norvancomycin and (b) Vancomycin on enantioselectivity of (*R*)- and (*S*)-CIT.

**Figure 4:**
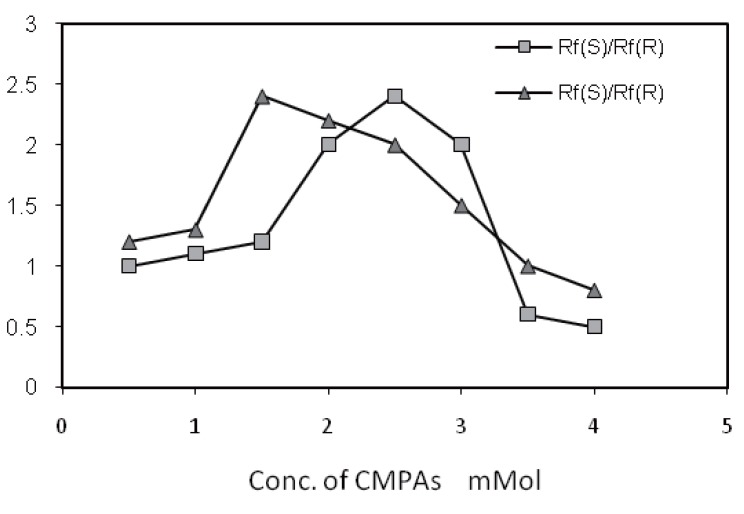
Effect of concentration (m Mol) of CMPAs. (a) Norvancomycin 1.5 m Mol and (b) Vancomycin 2.5 mMol on enantioselectivity of (*R*)- and (*S*)-CIT.

The concentration of NORV in the mobile phase could influence both the significant resolution and the enantioselectivity. NORV and VANC as chiral additive showed similar abilities of enantioselectivity as shown in (Fig. 2). Significant Rf values are shown to increase with an increase in NORV concentration from 1-1.5 mM followed by a slight decrease at NORV concentration between 2.0 and 4.0 mM. Overall, good enantioselectivity and satisfactory resolution of spots were simultaneously found at concentration level of 1.5 mM NORV or 2.5 mM VANC as shown in (Fig. 3).

(Fig. 4: 1) represent real picture of TLC-chromatogram for enantiomeric resolution of (*R, S*)-CIT and its related substances in mobile phase containing 1.5 or 2.5 mM chiral selector, where (*R, S*)-CIT were resolved into two spots of *R*-enantiomer R_f_ 0.33 and *S*-enantiomer R_f_ 0.85. (Fig. 4: 2) shows real picture of TLC-chromatogram for resolution of (*R, S*)-CIT and its related substances in the same mobile phase without presence of chiral selector, where (*R, S*)-CIT were resolved into one spot only the *S*-enantiomer R_f_ 0.85.

**Figure 5:**
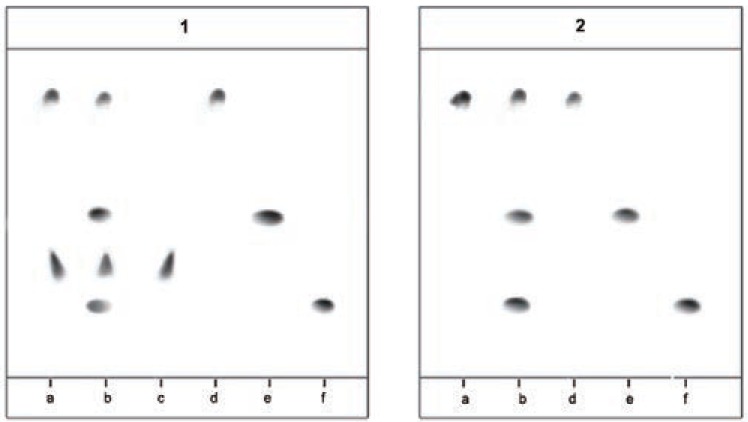
Thin layer chromatogram show resolution of (a) *R, S*-citalopram 16 μg/10 μl; (b) mixture of *R, S*-citalopram 16 μg/10 μl and its related impurities; citalopram citadiol 4.4 μg/10 μl, and citalopram N-oxide 4.4 μg/10 μl; (c) *R*-citalopram 8 μg/10μl in 1); (d) escitalopram 8 μg/10 μl; (e) citadiol 4.4 μg/10 μl, and (f) citalopram N-oxide 4.4 μg/10 μl using mobile phase; acetonitrile-methanol-water (15:2.5:2.5v/v) at 25 ± 2 C. 1) With chiral selector 1.5 mM norvancomycine or 2.5 mM vancomycine; 2) Without chiral selector.

**Figure 6:**
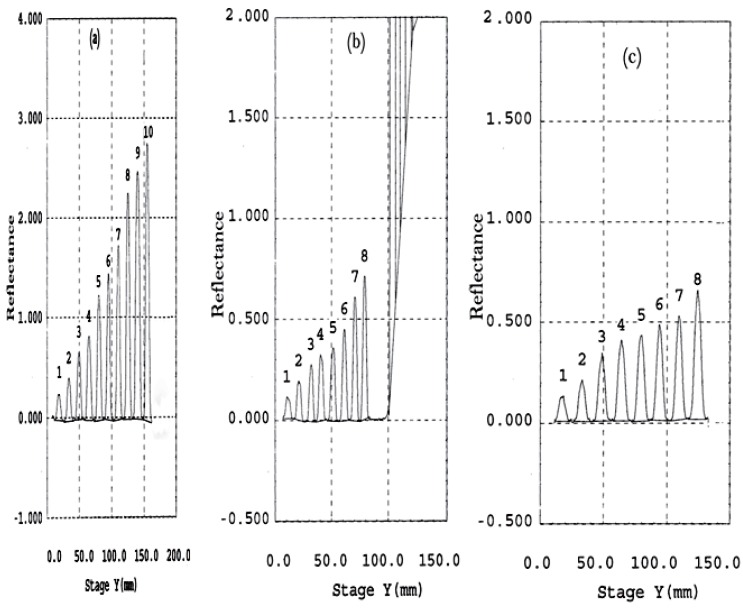
Densitometric scanning profile for TLC–chromatogram of different concentrations of (a) (*R*)-citalopram or (*S*)- citalopram (escitalopram) 0.2-16.8 μg/10 μl, (b) citalopram citadiol 0.1-4.4 μg/10 μl, (c) citalopram N-oxide 0.1-4.4 μg/10 μl, using mobile phase; acetonitrile-methanol-water (15:2.5:2.5 v/v) ,containing 1.5 mM norvancomycine at 25 ± 2 C.

Different scanning wavelength was tried; peaks at 239 nm (λ_max_ of CIT) gave higher sensitivity. Densitometric scanning profile of TLC-chromatogram for all compounds at different concentrations was shown in (Fig. 5). TLC scanning profile of (*R*)-CIT or (*S*)-CIT (escitalopram) and its related impurities; CIT-C and CIT-N over the corresponding linearity range at 239 nm have been represented in (Fig. 6).

### System Suitability tests

According to USP (20), system suitability tests are an integral part of the chromatographic separation methods in the course of optimizing the conditions of the proposed method. In the TLC-method system suitability tests are used to verify that the resolution and repeatability of the system were adequate for the analysis performance. Different parameters affecting the enantiomeric separation were studied. The parameters of this test are relative retardation (resolution factor) and RSD% of 5 repeatable determinations of peak areas and reproducibility of Rf values for the separated compounds. The results of these tests prove good resolution of the compounds (Table 3).

**Table 3:** System sutability test for the proposed TLC method for the determination of *S*-citalopram, citalopram-citadiol, *R*-citalopram and citalopram-Noxide

Parameters	*S*-CIT	CIT-C	*R*-CIT	CIT-N

R_ret_^a^	1.89	1.36	1.50	-
RSD %^b^				
Peak areas	0.57	0.80	0.73	0.66
R_f_ value*s*	0.08	0.03	0.04	0.02

a Relative retardation ratio (resolution factor);

b RSD% of 5 repeatable determinations.

### Quantification, accuracy and precision

Standard calibration curves were prepared by separately preparing series of different concentrations of the cited compounds and applying the suggested procedure under the optimized chromatographic conditions. The linearity of the calibration curves were validated by the high value of correlation coefficients. Plots of peak areas × 10^-2^ versus the concentrations μg/10 μl^-1^ of the analyte within the respective ranges are shown in (Table 4). The data were subjected to statistical analysis using the corresponding regression equations. The sensitivity of the proposed TLC method was determined with respect to LOD and LOQ. The lowest amounts of (*R, S*)-CIT which could be detected or quantified were calculated and found to be 0.08 and 0.25 μg/10 μl of (R) - and (*S*)-citalopram ,respectively. The LOD and LOQ of relater impurities; CIT- C and CIT- N were 0.06 and 0.19 μg/10 μl Table 3. Precision of the proposed method was determined by repeatability (intra-day) and intermediate inter-day assay and the results obtained are recorded in (Table 4) where inter and intra-assay precision of RSD≤2% are provided. The regression equations of these calibration graphs were utilized for the determination of concentrations of the cited compounds in laboratory prepared mixtures and tablets.

**Table 4:** Results obtained by TLC-method for determination of (*S*)- and (*R*)-citalopram in ternary mixture with its related substances citalopram citadiol, and citalopram N-oxide

Parameters	ESC	R-CIT	CIT-C	CIT-N

Range μg/10 μl	0.2-16.8	0.2-16.8	0.1-4.4	0.1-4.4
Linearity				
Slope	6.2992	6.3081	6.6406	5.8724
Intercept	4.3627	3.0419	2.4203	2.85337
(r)	0.9995	0.9994	0.9994	0.9996
*Accuracy*^a^ ± RSD%				
Drug substance	99.51 ± 0.61			
Drug product	98.23 ± 0.92			
Authentic added	100.33 ± 0.75			
*Precision* ± RSD%				
Repeatability^b^	0.45	0.52	0.61	0.88
Intermediate^c^	0.92	0.94	1.01	1.10
Specificity ± SD%	99.71 ± 0.78	99.45 ± 0.66	99.19 ± 0.73	99.87 ± 0.73
LOD^d^ μg/10 μl	0.06	0.06	0.03	0.03
LOQ^d^ μg/10 μl	0.20	0.20	0.10	0.10

a Average of five determination (n=5);

b Repeatability (n=3), average of three different concentrations repeated three times day;

c Intermediate precision (n=3), average of three concentrations repeated three times in three successive days;

d Limit of detection and limit of quantitation, average of five determination (n=5).

Successful resolution of compact spots of (*R, S*)-CIT and its related substances was achieved with significant R_f_ values ± 0.02 of 0.33, 0.85, 0.45 and 0.22 for (*R*)-CIT, (*S*)-CIT, CIT-C and CIT-N, respectively. The method was able to determine the purity of (*R, S*)-CIT or (*S*)-CIT (ESC) in the presence of up to 150% of its related substances without interference. The % recovery values ± RSD of Escitalopram, *R*-Citalopram, Citalopram-C, and Citalopram-N are presented in (Table 5).

**Table 5:** Results of analysis of laboratory-prepared mixtures containing different percentages of the (*S*)-enantiomer (Escitalopram) and related substances by the proposed method

Amount spotted	% Recovery^a^			
	Escitalopram	R-Citalopram	Citalopram-C	Citalopram-N

0.05	98.90	98.45	98.64	99.11
10	99.10	99.20	98.95	100.40
50	100.45	99.58	100.45	99.65
100	100.60	100.20	98.75	100.85
150	99.50	99.80	99.10	99.33
Mean ± RSD	99.71 ± 0.78	99.45 ± 0.66	99.19 ± 0.73	99.87 ± 0.73

a Mean ± RSD for three determinations.

Accuracy studies were done by analyzing five concentrations within the limiting linearity ranges with a good percentage of mean recovery (99.70 ± 0.85 and 99.51 ± 0.61) for (*S*)-CIT and ESC, respectively, indicating accurate results Table 5. Statistically comparison of the results was performed with regard to accuracy and precision using Student’s-test and F-ratio at the 95% confidence level, and there were no significant difference between the proposed method and the reported one (13) (Table 6).

**Table 6:** Statistical comparison between the results of the proposed TLC-method and the reported one for the determination of *S*-citalopram and escitalopram in pure powder form

Parameters	Proposed TLC method		Reported TLC method^a^	
	*S*-CIT	ESC	*S*-CIT	ESC

Mean^b^	99.70	99.51	99.00	99.15
RSD	0.85	0.61	1.13	1.08
SE	0.38	0.27	0.51	0.48
Variance	0.72	0.37	1.25	1.14
*t*-test	1.10	0.65	(2.3)^c^	(2.3)^c^
*F*-value	1.74	3.08	(6.4)^c^	(6.4)^c^

a Reported TLC method for assay of *S*-citalopram using acetonitrile-water (17:3), and 1 mM brucine sulphate as a chiral selector (13);

b Average of five determination (n=5);

c Figures between parentheses represent the corresponding tabulated values of *t* and *F* at *p*=0.05.

Also, the applicability of the procedure for estimation of Depram and Estikan tablets was validated using standard addition technique as a check of accuracy. The standard addition recoveries were carried out by adding three different concentration levels (0.4, 1, 2 μg/10 μl of (*R*)-CIT enantiomer or (2, 4, 8 μg/10 μl of (*S*)-CIT enantiomer to the powdered tablets. No interference from the formulation excipients such as avicel pH102, lactose, cross carmelose sodium, colloidal silica dioxide, etc was observed. The results obtained indicate good recovery (Table 7).

**Table 7:** Results of application of standard addition technique for the determination of *R, S*-CIT in Depram tablets (40 mg) and ESC in Estikan tablets (20 mg)

Tablets (μg/10 μl)	Authentic added^a^ (μg/10 μl)	Recovery %		
		(*R, S*) citalopram		Authentic added^b^ ± RSD

Depram tablets		R-CIT	S-CIT	
4	*R*-enantiomer	48.38	49.16	100.59
6	*S*-enantiomer	49.30	50.25	99.10
Mean ± RSD				99.45 ± 1.02
Estikan tablets		Escitalopram		
4	*R*-enantiomer	97.00		99.32
6	*S*-enantiomer	98.50		99.00
Mean ± RSD		98.23 ± 0.92		100.33 ± 0.75

a Concentration of *R*-enantiomer added at three different levels 0.4, 1, 2 μg/10 μl and *S*-enantiomer added at three different levels at 2, 4, 8 μg/10 μl;

b Mean of three determinations.

Robustness is a test of the method which remains unaffected by small variations in the method conditions and expressed as %RSD, and it is an indication of the method reliability as shown in (Table 8).

**Table 8:** Robustness testing

Parameters	Recovery^a^ % ± RSD	

	*S*-CIT	ESC
1. Mobile phase composition	101.67 ± 1.29	100.67 ± 1.21
2. Plate size	101.25 ± 0.89	101.54 ± 0.62
3. Acetonitrile in the mobile phase	100.54 ± 0.85	99.36 ± 0.96
4. Glass chamber size	98.90 ± 0.67	98.68 ± 0.85
5. Developing TLC distance	101.64 ± 1.20	100.85 ± 1.31
6. Concentration of chiral selector	98.40 ± 0.78	98.75 ± 0.69
7. pH of mobile phase (± 0.2)	100.12 ± 0.86	99.35 ± 0.92
8. Chamber saturation time	99.01 ± 0.86	98.90 ± 1.23

a Average of five determination (n=5).

### Analysis of the related substances

The USP (20) determine the chromatographic purity limiting individual impurity to 0.1% *w/w* and the sum to 0.5% *w/w*. The related substances were determined by spotting higher concentrations of the drug in order to detect and quantify them (10, 12). Different batches of (*R, S*)-CIT or ESC were investigated to determine the actual concentrations of the related substances relative to 10 mg/ml of (*S*)-CIT or ESC. The proposed procedure allows the determination of the related substances in the bulk drug and tablets (Table 9). The results indicate good percentage recoveries (48.83 ± 0.58 – 51.65 ± 0.64% of (*S*)-CIT and 48.60 ± 0.41 – 49.93 ± 0.65% of (*R*)-CIT. Moreover 0.20 ± 0.03 % of CIT-N and 0.11 ± 0.06 % of CIT-C as related substances could be detected in bulk drug. Depram tablets and citalo tablets contained only very low levels of both related substances (Table 9).

**Table 9:** Results of analysis of the related substances % *w/w* relative to 10 mg/ml (*S*)-citalopram and (escitalopram) in bulk drug and fomulations

Sample claimed value^a^ (mg)	Found (μg/10 μl)		Recovery % ± RSD^e^		Impurities % w/w ± RSD^e^	
	*S*-CIT	R-CIT	*S*-CIT	R-CIT	CIT-N	CIT-C

(*R, S*)-Citalopram						
Bulk drug	10.10	9.95	50.50 ± 0.75	49.75 ± 0.52	0.20 ± 0.03	0.13 ± 0.06
Depram tablets 40mg	19.53	19.97	48.83 ± 0.58	49.93 ± 0.65	0.09^b^ ± 0.02	0.08 ± 0.02
Citalo tablets 20mg	10.33	9.72	51.65 ± 0.64	48.60 ± 0.41	0. 18 ± 0.03	0.14 ± 0.03
Escitalopram						
Bulk drug	9.91	0.09	99.10 ± 1.89	0.90 ± 0.02	ND^c^	ND^c^
Cipra-Pro tablets 10mg	10.14	0.12	101.40 ±1.59	1.20 ± 0.03	0.10 ± 0.03	0.10^d^ ± 0.04
Estikan tablets 20mg	20.50	0.28	102.50 ± 0.98	1.41 ± 0.06	0.03^d^ ± 0.02	0.11^d^ ± 0.05

a The amount of drug in tablets is expressed as label claimed value;

b The amount of impurities are relative to a concentration of (*S*)-citalopram or escitalopram(10 mg/ml);

c Not detected;

d Below LOQ;

e Mean ± RSD.

In addition, the peak purity of ESC allows the assay of the bulk drug and tablets in the presence of its related substances. The percentage recoveries were 99.10 ± 1.89% – 102.50 ± 0.98 of ESC, and 0.9% of (*R*)-CIT could be detected in ESC bulk drug. CIT-N and CIT-C could not be detected in any samples of ESC. Cipra-Pro tablets and Estikan tablets contained about 101.40 ± 1.59% – 102.50 ± 0.98 % of ESC 1.20 ± 0.03 % – 1.41 ± 0.06 % (*R*)-CIT could be detected in the tablets. The related substances CIT-N and CIT-C were below LOQ. The assay of related substance was validated based on a final concentration of (10 mg/ml (*S*)-CIT or ESC. The assay results of (*R, S*)-CIT and ESC in bulk drug and tablets are expressed as label claimed value (12) as shown in (Table 9).

## CONCLUSION

The present work describes a newly enantioselective TLC-method for the simultaneous determination of *R*- and *S*-CIT using norvancomycin or vancomycin which enable chiral recognition via formation of host-guest inclusion complexes. All the drug and its related substances are closely similar in their structure and have completely overlapped spectra, therefore the ability to separate and quantify the active ingredients as well as the related substances without interference is one of the favorable advantages of the method. The procedure would be useful for stability investigation of CIT in synthesis, pharmaceutical preparation, quality control and can be extended for routine analysis.
